# A Phase I/II trial comparing autologous dendritic cell vaccine pulsed either with personalized peptides (PEP-DC) or with tumor lysate (OC-DC) in patients with advanced high-grade ovarian serous carcinoma

**DOI:** 10.1186/s12967-019-02133-w

**Published:** 2019-11-26

**Authors:** Apostolos Sarivalasis, Caroline Boudousquié, Klara Balint, Brian J. Stevenson, Philippe O. Gannon, Emanuela Marina Iancu, Laetitia Rossier, Silvia Martin Lluesma, Patrice Mathevet, Christine Sempoux, George Coukos, Urania Dafni, Alexandre Harari, Michal Bassani-Sternberg, Lana E. Kandalaft

**Affiliations:** 1 Department of Oncology, Centre Hospitalier Universitaire Vaudois and University of Lausanne, Lausanne, Switzerland; 2 Swiss Institute of Bioinformatics, 1015 Lausanne, Switzerland; 3 Women-Mother-Child Department, Service of Gynecology, University Hospital of Lausanne, Lausanne, Switzerland; 4 Department of Pathology, University Hospital of Lausanne, Lausanne, Switzerland; 5 Ludwig Institute for Cancer Research, University of Lausanne, Lausanne, Switzerland; 6 Laboratory of Biostatistics, School of Health Sciences, National and Kapodistrian, University of Athens, Athens, Greece

**Keywords:** Ovarian cancer, Dendritic cell vaccine, Neoantigen, Neoepitope, Cancer immunotherapy, Cyclophosphamide

## Abstract

**Background:**

Most ovarian cancer patients are diagnosed at a late stage with 85% of them relapsing after surgery and standard chemotherapy; for this reason, new treatments are urgently needed. Ovarian cancer has become a candidate for immunotherapy by reason of their expression of shared tumor-associated antigens (TAAs) and private mutated neoantigens (NeoAgs) and the recognition of the tumor by the immune system. Additionally, the presence of intraepithelial tumor infiltrating lymphocytes (TILs) is associated with improved progression-free and overall survival of patients with ovarian cancer. The aim of active immunotherapy, including vaccination, is to generate a new anti-tumor response and amplify an existing immune response. Recently developed NeoAgs-based cancer vaccines have the advantage of being more tumor specific, reducing the potential for immunological tolerance, and inducing robust immunogenicity.

**Methods:**

We propose a randomized phase I/II study in patients with advanced ovarian cancer to compare the immunogenicity and to assess safety and feasibility of two personalized DC vaccines. After standard of care surgery and chemotherapy, patients will receive either a novel vaccine consisting of autologous DCs pulsed with up to ten peptides (PEP-DC), selected using an agnostic, yet personalized, epitope discovery algorithm, or a sequential combination of a DC vaccine loaded with autologous oxidized tumor lysate (OC-DC) prior to an equivalent PEP-DC vaccine. All vaccines will be administered in combination with low-dose cyclophosphamide. This study is the first attempt to compare the two approaches and to use NeoAgs-based vaccines in ovarian cancer in the adjuvant setting.

**Discussion:**

The proposed treatment takes advantage of the beneficial effects of pre-treatment with OC-DC prior to PEP-DC vaccination, prompting immune response induction against a wide range of patient-specific antigens, and amplification of pre-existing NeoAgs-specific T cell clones.

*Trial registration* This trial is already approved by Swissmedic (Ref.: 2019TpP1004) and will be registered at http://www.clinicaltrials.gov before enrollment opens.

## Background

Ovarian cancer (OC) is the primary cause of gynecologic cancer-related deaths, with more than 300,000 expected new cases, and more than 190,000 estimated deaths worldwide in 2020 [1]. New treatment approaches for ovarian cancer care are urgently needed, as current therapies, including cytoreductive surgery and platinum-based chemotherapy, do not cure most patients with advanced epithelial ovarian cancer [2].

The host immune system can recognize and target ovarian cancer [3], in which a variety of tumor-associated antigens (TAAs) have been demonstrated (HER-2/neu [4]; p53 [5, 6]; the folate binding protein [7], sialyated TN [8], MUC-1 [9], NY-ESO-1 [10] or mesothelin [11] among others). Furthermore, patients presenting TILs in their ovarian cancer tissue show longer progression-free and overall survival (PFS and OS) [3], indicating that tumor growth is under the surveillance of the immune system, and suggesting that ovarian cancer is a good candidate for immunotherapy [12]. Nevertheless, the efficacy of immunotherapy may be decreased by the involvement of various mechanisms of immune evasion in the tumor microenvironment including high expression of PD-L1 [13], production of IDO [14], recruitment of regulatory T-cells (Tregs) [15, 16], or local and systemic dysfunction of plasmacytoid dendritic cells (pDCs) [17].

### Cancer vaccines

Cancer vaccines are intended to “educate” the patient’s own immune system to generate effector T-cells specifically for tumor cells to be detected and destroyed. A tailored cancer vaccine aims to target multiple patient-specific tumor antigens and reduce side-effects by protecting normal tissue and keeping tumors under immune memory regulation for as long as possible [18]. Dendritic cell (DC)-based vaccines are a particularly attractive option for immunotherapy, due to their low toxicity profile, lack of invasive procedures and their potential to induce long-term effects through immunological memory [19]. DCs are unique immune cells responsible for processing and presenting cancer antigens, and are capable of initiating and regulating both innate and adaptive immunity [20]. DCs can present endogenous antigens as human leukocyte antigen (HLA) class I peptides and exogenous antigens as either HLA class II peptides or HLA class I peptides by cross presentation, thus effectively inducing antigen-driven T-cell responses. Monocyte-derived human DCs pulsed with TAAs have been extensively used for clinical therapies against malignancies [21]. Unfortunately, DC vaccines have demonstrated limited efficacy in patients with advanced recurrent disease [22]. Some promising results however suggest a need for further optimization, including combination of different immunotherapy technologies and multiple antigens.

Key factors leading to the poor immune response in ovarian cancer include lack of well-characterized tumor antigens, molecular heterogeneity, selective tumor antigen-loss (immuno-editing) and the immunosuppressive nature of the tumor microenvironment [23]. When vaccines target defined non-mutated self-antigens or shared antigens that are overexpressed in the tumor, vaccine efficacy is often low because T cell reactivity to self-antigens is naturally reduced due to central tolerance [24]. Alternatively, neoantigens (NeoAgs) that arise from somatic DNA alterations as a result of genetic instability are cancer-specific and can be strongly immunogenic [25]. NeoAgs are likely to be effective targets for tumor infiltrating T cells and can lead to successful immunotherapy treatments [26], hence synthetic vaccines targeting patient-specific NeoAgs can display increased efficacy against tumors with moderate or high mutation load. Three recent phase I studies using personalized NeoAg-based vaccines reported immunogenicity and interesting clinical safety and efficacy results [27–30].

### Whole tumor lysate vaccines

An alternative source of personalized antigens is the whole tumor lysate. In the case of ovarian cancer, tumor cells can be easily recovered by cytoreductive surgery and tumor antigens can be obtained directly from the patient’s own tumor cells by preparing a tumor lysate that contains both the TAAs and the private NeoAg without the hurdles of target identification and preparation [31]. The immunogenicity of whole-tumor antigen vaccines can be enhanced by different tumor lysate preparation methods [31, 32]. During the final culture stage, tumor lysate is loaded onto the DCs and then the DC vaccine is presented to the immune system via intranodal injection(s). The advantage of these whole tumor vaccines is that they target a whole range of antigens thus reducing the chance of tumor escape compared to single epitope vaccines. They are independent of HLA type and consist of epitopes for CD8+ cytotoxic T-cells (CTLs) and CD4+ T helper (Th) cells, leading to a more integral immune response [33]. Previous studies with DC vaccines loaded with whole tumor lysate have already demonstrated that patients with measurable disease, in cases such as non-Hodgkin’s lymphoma [34], melanoma [35], or renal cancer [36], may obtain clinical benefit. We also reported previously on a vaccine generated using autologous monocyte-derived DCs pulsed with autologous tumor lysate produced from tumor tissue dissociated into single cells, oxidized and lysed using freeze–thaw cycles [37]. Hypochlorous acid (HOCl) oxidization approach has previously been shown to be superior to ultraviolet B irradiation or freeze–thaw of tumor lysis, in terms of priming T cell responses against tumor antigens in vitro, and has shown promise in an OC preclinical model [37]. We also showed that it was feasible to produce oxidized lysate-pulsed DCs in five patients with OC and to perform intranodal injections of this vaccine [37], which consisted of ex vivo-cultured, autologous monocyte-derived DCs pulsed with oxidized autologous tumor lysate [38, 39].

In a phase I trial, the OC-DC vaccine was tested in 25 patients with platinum-treated, immunotherapy-naïve, recurrent ovarian cancer in which study the OC-DC treatment injected intranodally was found to be safe, feasible, able to induce T cell responses to autologous tumor antigen, and was associated with significantly prolonged survival [40]. In the cohort receiving the vaccine combined with cyclophosphamide and bevacizumab, the median progression free survival was 11.1 months compared with 4.1 months in the historical control population. Survival at 2 years was 100% for the vaccine plus bevacizumab/cyclophosphamide group, while it was only 40% for patients receiving vaccine and bevacizumab only, as well as for control patients receiving bevacizumab and cyclophosphamide (p = 0.011), confirming that adding cyclophosphamide to the vaccine could increase its effect. The 2-year OS rate for patients responding to treatment was 100%, whereas it was 25% for non-responders. Importantly, only patients with an immune response to whole tumor lysate or autologous tumor showed clinical benefit [40]. Upon analysis of the peripheral blood mononuclear cells (PBMCs) collected pre-vaccination and after five doses of induction vaccination, the frequency of tumor-reactive T cells in the peripheral blood was found to increase gradually over time. Furthermore, six patients displayed CD8+ responses on immunization against one or more neo-epitopes. This study was the first to show that vaccination with whole tumor lysate loaded DCs elicited a CD8 T cell response to antigens derived from private non-synonymous somatic tumor mutations. These results demonstrate that personalized vaccines using whole tumor lysate can enhance pre-existing immune responses to NeoAgs as well as rationalize the potential combinatorial use of whole tumor lysate vaccine followed by NeoAgs targeting to enhance the anti-tumor effect of vaccinations [40].

### NeoAgs in ovarian cancer

Early preclinical evidence suggested that ovarian cancer was unsuitable for NeoAg-specific vaccination [41]. Subsequent analysis of human ovarian cancer specimens, however, indicated that ovarian cancer tissues may express NeoAgs [42, 43] and that their expression is associated with better OS [44]. NeoAgs promise high specificity but are hard to identify because they are mostly patient-specific, and they are mainly rare events in a patient cohort. Personalized vaccine design requires the identification of each patient’s NeoAg tumor repertoire (“mutanome”), which has only been possible in recent years due to significant technological advancements such as next-generation sequencing (NGS), analysis of immunopeptidome by mass spectrometry (MS) and development of bioinformatical prediction tools [45–48]. Limitations concerning the availability of relatively large sample volumes requested (> 1 g of tissue) and the logistics involved in the operation of specialized and sophisticated MS instruments have so far hampered the integration of immunopeptidomics into routine clinical practice. The implementation of an individualized treatment concept based on the mutanome requires both highly interdisciplinary research and an innovative drug development process [49]. It is therefore unclear at this point, whether synthetic vaccines based on private antigens are going to be more effective than whole tumor lysate vaccines and could potentially replace them.

## Methods/design

### A novel approach to therapeutic vaccination with enhanced efficacy—PEP-DC Vaccine

**Fig. 1:**
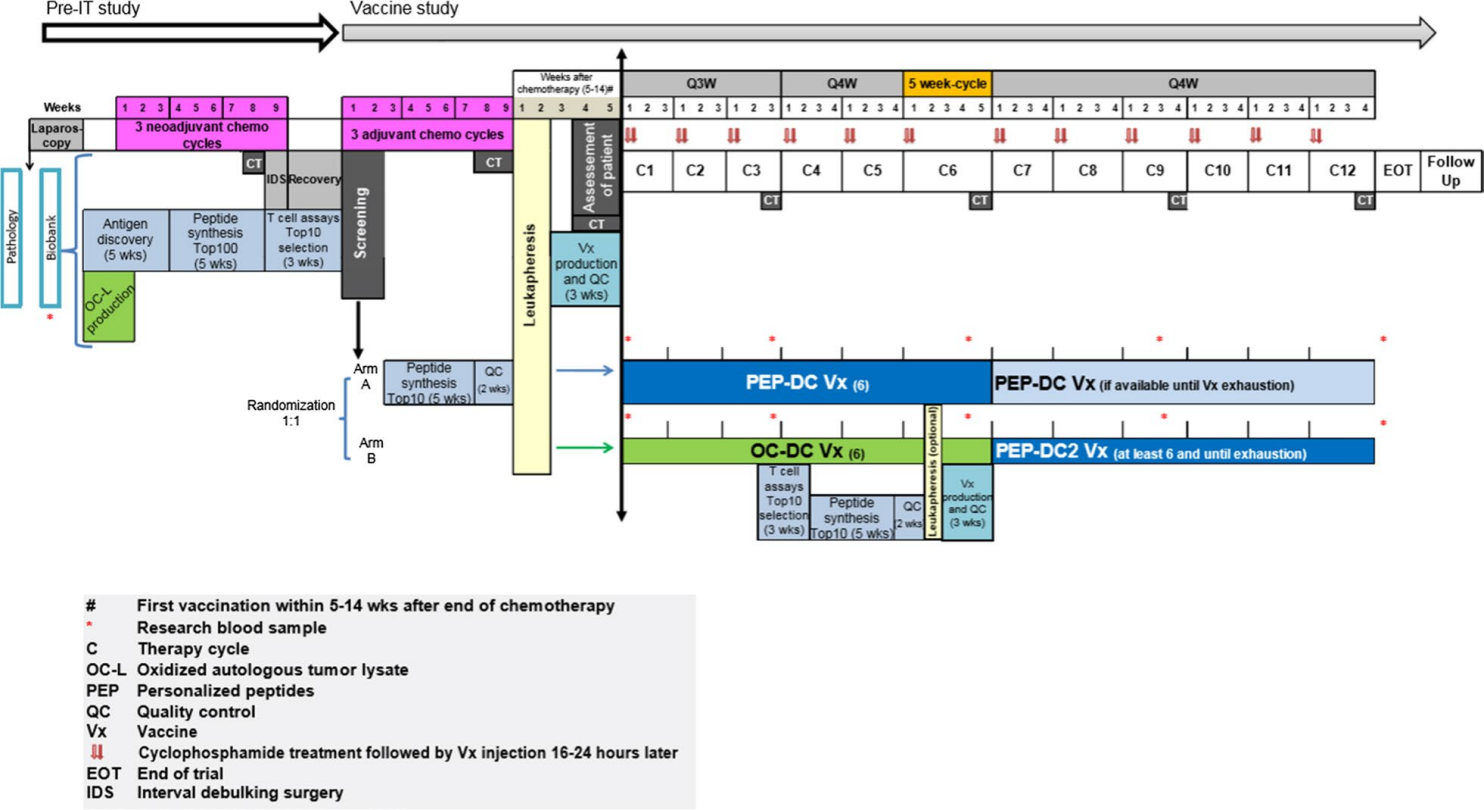
Clinical trial study design

Based on our previous OC-DC study observations, the specific recognition and targeting of tumor specific NeoAgs is a powerful way to further enhance the effectiveness of OC-DC vaccine. We will therefore use a personalized vaccine consisting of autologous monocyte derived DCs pulsed with personalized peptides (PEP-DC) combining tumor-specific NeoAgs and TAAs in patients with advanced ovarian cancer. In order to validate our hypothesis, patients will be randomized to receive either OC-DC or the novel PEP-DC vaccine during the initial part of this study; then the OC-DC arm will switch over to subsequently receive PEP-DC. We are therefore proposing a phase I/II, randomized two-cohort, single-center study to compare immunogenicity and assess the safety of personalized peptide pulsed DC vaccine (PEP-DC) alone, or oxidized tumor cell pulsed DC (OC-DC vaccine) followed by PEP-DC (PEP-DC2). After the comparative phase of vaccination (six cycles), all patients will be vaccinated with PEP-DC. All vaccines will be administered intranodally and applied in combination with cyclophosphamide in patients with OC (Fig. 1).

Based on the patient’s own tumor, epitopes for the PEP-DC vaccine will be determined and will be identified and produced from snapped-frozen tumor specimens and blood samples. The tumor specimens are required for the production of oxidized whole tumor lysate, for DNA and RNA extraction to complete NGS and for elution of the HLA binding peptides for MS based immunopeptidomics [50, 51]. The blood samples will be used for PBMC isolation and HLA-typing. PBMC-derived DNA will serve as the germline reference genome in order to identify personalized NeoAgs. Genomic variants affecting coding genes that are present in the tumor samples and absent from the corresponding blood samples are assumed somatic. The NGS data will be analyzed using our NeoDisc pipeline [52] to generate personalized reference databases for each patient. The MS-based immunopeptidomics data will be searched against the reference database in order to directly identify in vivo presented NeoAg as well as TAAs. In addition, based on these references, NeoAg binding to HLA-I and HLA-II molecules will be predicted in silico. Eventually, 100 personalized targets for each patient will be selected and redesigned as ~ 25mer peptides optimally spanning across the predicted ligands [52].

In order to narrow down the number of target antigens to maximum 10 per patient, we have set-up robust T-cell assays to test patient’s PBMCs, T cells and TILs for their reactivity to private peptides. Using the patients’ isolated CD4 and CD8 T-cells, we will perform functional validation of immune reactivity against the 100 candidate peptides and quantify it by IFNγ ELISpot assay. This additional step allows us to incorporate immunogenic antigens into the PEP-DC design to enhance the patient’s already existing anti-tumor immune response after vaccination, and hence, to increase the vaccine’s potential. Following this immunogenicity analysis, the targets list will be reduced to 10 target sequences and ‘enhanced quality’ peptides for the Top 10 targets will be ordered from Almac (Edinburgh, UK) for vaccine manufacturing. As a few peptides might fail synthesis, up to 10 targets will be eventually used. The Top 10 peptides selected a priori will be used to formulate the PEP-DC vaccine for Arm A patients. In Arm B, patients will first receive OC-DC vaccine then based on the immunogenicity analysis on PBMCs collected a priori and after three OC-DC vaccines (at the end of cycle 3) a personalized PEP-DC vaccine will be designed for them. The top 10 peptides determined at this time point (reflecting new immune responses induced by the preceding OC-DC vaccines) will be used to formulate the PEP-DC2 vaccine to be administered to patients in Arm B after six OC-DC administrations.

### A new dendritic cell vaccine closed system platform

OC-DC vaccine will be manufactured using oxidized whole tumor cell lysate derived from autologous tumor harvested during laparoscopy surgery or interval debulking surgery (IDS) pulsed onto autologous dendritic cells. The choice of dendritic cell maturation and the choice of tumor cell preparation are based on data previously published [53]. In this study, the vaccines’ components include agents for which safety has previously been shown to be acceptable [40].

PEP-DC vaccines will be manufactured and formulated at the GMP (good manufacturing practice) facility of the Center of Experimental Therapeutics (CTE, Lausanne) and the Centre Hospitalier Universitaire Vaudois (CHUV, Lausanne). After the Top 10 peptides have been identified and manufactured, patients will undergo leukapheresis for generation of DCs. Autologous monocytes will be enriched in a CliniMACS Prodigy (Miltenyi Biotec) system that is a GMP-compliant closed system, allowing for standardized and reproducible cellular processing across multiple instruments. The leukapheresis bag will be attached using sterile tubing set to the CliniMACS Prodigy device, and with the predefined LP-14 enrichment program and the CD14 reagent (magnetic beads, Miltenyi Biotec), CD14+ monocytes will be purified by positive enrichment. Purified monocytes will be differentiated into immature monocyte-derived DC (iDC) by a 5 days culture in the presence of clinical grade IL-4 and GM-CSF. iDC will then be loaded with a mix of the Top 10 peptides overnight and matured/activated for 6 to 8 h on day 6 using a maturation cocktail consisting of clinical grade monophosphoryl lipid A (MPLA) and IFNγ. Finally, cells will be harvested and cryopreserved as vaccine doses, comprising 5-10 x10^6^ cells per dose. For each injection of the PEP-DC vaccine, one dose will be thawed, washed and resuspended in NaCl 0.9% supplemented with 1% human albumin before being transferred into syringes and stored at 2–8 °C until administration to patients as intranodal injection.

### Using low-dose cyclophosphamide to enhance immune response

In a study of 104 ovarian cancer patients, regulatory T cells (Treg) have been shown to contribute to growth of human tumors in vivo [15]. It has been demonstrated that low-dose cyclophosphamide, when used in combination with immunotherapy, can reduce Treg numbers and impair their function without eliminating other immune cells [54–56], thus creating a favorable environment for greater efficacy and immune response. For instance, pre-treatment with intravenous low-dose cyclophosphamide (300 mg/m^2^) improved immunogenicity of a p53-synthetic long peptide vaccine in patients with recurrent OC [57]. Tanyi et al. [58] assessed OC-DC vaccine either alone (cohort 1, n = 5) or in combination with bevacizumab (cohort 2, n = 10), or bevacizumab plus low-dose intravenous cyclophosphamide (cohort 3, n = 10) in ovarian cancer patients until disease progression or exhaustion of vaccine doses. Low-dose cyclophosphamide (200 mg/m^2^) was administered about 24 h before each vaccine dose, and bevacizumab (10 mg/kg) was administered on the day of vaccination. The survival observed in the cohort with cyclophosphamide was longer than previously reported in this population with bevacizumab-based biological combinations [59, 60]. Consistently, in our study we will administer intravenous cyclophosphamide at a dose of 200 mg/m^2^ within 24 h prior to each vaccination.

### Clinical study design

This investigator-initiated trial will be conducted at the Department of Oncology at the CHUV. We propose a Phase I/II, randomized, two-cohort, single-center study in ovarian cancer patients to compare immunogenicity and assess the safety of PEP-DC alone (Arm A) or OC-DC vaccine followed by PEP-DC2 (Arm B); vaccines will be administered intranodally and in combination with cyclophosphamide in all cases. We consider that vaccination with synthetic vaccines (PEP-DC and PEP-DC2) developed on the basis of our integrated tumor antigen discovery pipeline is feasible, and can produce specific immune responses when applied in combination with low dose cyclophosphamide against mutated peptides and other private tumor antigens. The OC-DC vaccine therapy is expected to activate and expand T-cells that recognize both the NeoAg and shared tumor antigens, and to correlate with clinical benefit. Furthermore, we expect OC-DC priming to boost PEP-DC vaccination, as we hypothesize that a priori prediction of relevant immunogenic peptides is currently not optimal, and that upfront vaccination with whole tumor lysates may significantly enhance the detection of immunogenic antigens in in vitro T cell assays, and thus assist in the subsequent development of synthetic personalized vaccines. A randomization of 1:1 to arm A (PEP-DC) or arm B (OC-DC followed by PEP-DC2) will be performed to reduce potential selection bias on outcome.

#### Study objectives

The primary objective of the study is to determine the immunogenicity of PEP-DC vaccine compared to the OC-DC vaccine followed by PEP-DC2 vaccine, specifically its effect on eliciting/enhancing T-cell responses to private epitopes detected by our antigen identification algorithm. The secondary objectives of the study include the assessment of feasibility, safety, overall survival (OS), time to progression and disease-free survival rates at 12, 24, and 36 months in the two arms, using both RECIST 1.1 and CA 125 GCIG criteria. As an exploratory objective, we will determine whether a priori PEP-DC vaccine and OC-DC vaccine followed by PEP-DC2 vaccine elicit epitope spreading.

#### Eligibility criteria and number of patients

Informed consent will be obtained from all participants. Eligibility criteria require patients to be 18 years or older, with high grade serous ovarian cancer (HGSOC) at FIGO stage III or IV who completed either primary debulking surgery (PDS) or interval debulking surgery (IDS) without residual disease (R0), and have received either at least 3 cycles of chemotherapy in the per-operative chemotherapy and IDS design, or 6 cycles of platinum-based chemotherapy after PDS. A total of 16 evaluable patients (8 patients in each arm) is required to enter this small, randomized Phase I/II trial, in order to have adequate power (at least 80%) for testing, at an alpha of 0.05 for differences between the immunogenicity in the two arms.

#### Regimen

We anticipate that the majority of patients will undergo IDS procedure while a small fraction of them will receive paclitaxel and carboplatin adjuvant chemotherapy after PDS according to the institution’s SoC treatment algorithms. Prior to surgery, ovarian cancer patients will be offered the option for collection of surgically debulked ovarian tumor tissue and blood sample that will be processed in the laboratory for antigen identification under a dedicated research protocol.

After enrollment, patients who completed IDS without residual disease will be randomized into either Arm A or Arm B while starting their adjuvant chemotherapy (Fig. 1). After the last chemotherapy cycle, all randomized patients must undergo a 10–15 L apheresis to harvest PBMCs that will be transferred to the Tumor Processing Facility at the CTE for DC manufacturing. In both trial arms, cyclophosphamide will be administered intravenously at a dose of 200 mg/m^2^ 1 day before to each vaccination (W1D1 of each cycle). Each dose of any DC vaccine (PEP-DC, OC-DC or PEP-DC2) will contain 3–10 × 10^6^ antigen-loaded autologous DCs in a total volume of 1 mL and will be delivered as two intranodal injections, half dose in each thigh under ultrasound guidance (W1D2 of each cycle).

Patients randomized to Arm A will receive six personalized PEP-DC vaccinations, the first three every 3 weeks on Day 2, 23 and 44 (± 3 days), followed by at least three more PEP-DC vaccinations administered every 4 weeks (± 3 days), except in cycle 6 that will last 5 weeks (± 3 days). Additional PEP-DC vaccines may be administered every 4 weeks (± 3 days) if available, until exhaustion of vaccine doses or progression of the disease, whichever occurs earlier.

Patients randomized to Arm B will first receive six OC-DC vaccines on Q3 W (± 3 days) to induce antitumor immune responses, the first three of them 3 weeks apart and from the fourth OC-DC injection onward every 4 weeks (± 3 days), or 5 weeks (6th cycle). Based on the results of the immunogenicity assay (using blood samples collected at baseline and after the third OC-DC cycle), the Top 10 antigen targets to be incorporated in the PEP-DC2 will be re-prioritized to allow inclusion of tumor antigenic targets against which the first three OC-DC vaccines induced specific T cell responses. After the sixth OC-DC vaccination, patients in Arm B may undergo a second leukapheresis during weeks 18–19 to produce their PEP-DC2 vaccine. Then, patients will receive at least six personalized PEP-DC2 vaccines Q4 W (± 3 days) until exhaustion of vaccine doses or progression of the disease, whichever occurs earlier.

#### Safety, laboratory and imaging assessments

The clinical tumor response will be assessed by serum CA-125 measurement, and by computed tomography (CT) scans of chest, abdomen and pelvis. A 5-year follow-up counting from the first DC vaccine injection is planned. Blood samples will be collected for all patients in both arms for the purpose of immune monitoring and translational research at screening (for prioritization of Top 10 targets), before the 1st vaccine (C1W1D1), after the 3rd (C3W3) and 6th (C6W3-W5) OC-DC vaccine, then every 12 weeks ± 7 days, at EOT and every 3 months in the first year as well as and every 6 months during the second year.

#### Translational analysis

We will test the immunogenicity of the top 100 tumor antigens (specific to each patient) in both trial arms using peripheral blood samples from screening (before vaccine), before cycle 3, at cycle 6 and 9, as well as at EOT in both study arms to detect any improvement or change in antigen-specific immune response. We will conduct immune landscape analysis of tumor infiltrating immune cells using multiplexed immunohistochemistry (mIHC) on tumor samples to understand the interactions between tumor cells and the immune system within the tumor microenvironment. T cells and immune cells will be quantified by using lineage specific markers combined with functional markers, to assess the activation and differentiation status. PBMCs or TILs recognizing the TAAs NeoAgs will also be isolated and their TCR sequenced. Furthermore, tetramers against TAAs and NeoAgs will be synthesized and multicolor flow cytometry will be used to perform an extensive immunophenotyping of specific CD4+ and CD8+ cells in TILs and PBMCs collected at different time points. Flow cytometry analyses will include markers to assess T cell activation, inhibition, differentiation and functionality status. In parallel, mass cytometry (CyTOF) will be used to quantify, profile and phenotype their activation and functional status as well as other immune/stroma populations. For investigation of immune fitness, patients’ PBMCs will be interrogated with sets of TLR antagonists, antigens and mitogens in in vitro functional assays to quantify the capacity of immune cells to respond to different stimuli.

#### Statistical methods

Safety in both cohorts will be assessed using NCI Common Terminology Criteria for Adverse Events (CTCAE v5.0) from registration until 30 days after the last vaccine injection. Efficacy time-to-event endpoints will be evaluated separately by RECIST 1.1 and CA 125 GCIG criteria in the two treatment arms, based on the Kaplan–Meier method. The safety population includes all evaluable subjects who received at least one vaccine dose. An immunogenicity scoring will be determined as follows: for each of the 100 pre-determined peptides, a score will determine for how many peptides immunogenicity in T-cells based assays is newly detected (shift from undetectable to detectable) or increased (the frequency of T-cell directed against the epitope is increased by ≥ twofold). Immunogenicity will be compared between the two vaccine arms, at least in two time-points; first at the end of the third cycle and second after the sixth vaccine dose.

## Discussion

DC-based vaccines were introduced decades ago to increase the immunogenicity of cancer vaccines and since then have been administered to numerous patients with diverse tumor types. They have a favourable toxicity profile and are well-tolerated. As they have shown moderate efficacy in the past, we have chosen to apply concomitant immuno-modulation in the form of intravenous low-dose cyclophosphamide, which we have already tested in combination with intranodal OC-DC vaccine in the advanced ovarian cancer population [40]. Intranodal injection of DC allows administration of a defined quantity of DCs directly to the site of T-cell sensitization; it also allows the peak IL-12 secretion to be synchronized with their proximity to T cells, where IL-12 can exert its full effects during antigen presentation [61].

When tumor samples are available for vaccine preparation, different choices may be available for cancer vaccine design and the choice of the target antigen is critical. Personalized vaccines targeting NeoAgs emerge as a promising approach. NeoAgs are more likely to elicit strong T-cell responses, because T cell tolerance does not hamper their immunogenicity, and consequently epitope spreading and a broad anti-tumor immune response. As a downside, the process of sequencing, immunopeptidomics analysis, peptide manufacturing and GMP manufacturing of NeoAg vaccines is long and expensive [26], although costs may decrease as a result of technological improvements. Currently it is not clear whether NeoAgs are superior targets compared with TAAs or tumor cell lysates and we believe that vaccinating first with OC-DC will lead to the activation of specific T cells against NeoAgs included in the PEP-DC vaccine, and hence will improve its efficacy.

Despite the rapid progress, the future development of DC-based cancer vaccines for wide-ranging clinical applications remains difficult. It is costly and is logistically difficult to be applied to every patient and be widely available. However, if this therapy proves to be effective, it would not be impossible to deliver it to all patients; very similarly to engineered T cells, which have become readily available given the recent entry of the pharmaceutical industry to this arena [62]. Furthermore, alternative APC platforms are currently under evaluation by many investigators worldwide. Another challenge is to assess whether DC vaccines can be integrated with SOC chemo or radiotherapy in an adjuvant combinatorial treatment setting for different types of cancer. The combination of such approaches will be a major advancement in cancer vaccinology, enabling the development of vaccines with enhanced therapeutic efficacy to improve the outcome of patients.

## Conclusions

We propose a new therapeutic approach to harness antitumor immunity against ovarian cancer, which is innovative in many respects. First, it translates a novel concept of NeoAg vaccine delivery from laboratory to the clinic, and implements a new personalized and agnostic epitope discovery pipeline, that relies on advanced biocomputational methodologies, exome and RNA sequencing, mass spectrometry based immunopeptidomics, and validation of the potential epitopes through functional cell-based assays. This provides a highly specific target repertoire for immunotherapies and increases the chances of inducing a significant and effective immune response against established tumors, and the potential for improved efficacy compared to previous vaccines. Second, this study is the first attempt to use NeoAg-based vaccines in ovarian cancer, specifically in the adjuvant setting. The proposed therapy incorporates the beneficial effects of pre-treatment with OC-DC before PEP-DC vaccination to induce epitope spreading, immune response against a wide range of patient-specific antigens and to enhance pre-existing NeoAg-specific T cell clones. Third, there was no previous study comparing an autologous DC vaccine targeting NeoAgs to tumor lysate vaccines in the adjuvant setting in ovarian cancer. Collectively, this approach is novel for ovarian cancer, a condition in dire need of new therapies.

## Abbreviations

CD: cluster of differentiation
CHUV: Centre Hospitalier Universitaire Vaudois (Lausanne)
CT: computed tomography
CTCAE: Common Terminology Criteria for Adverse Events
CTE: Center for Experimental Therapeutics (at CHUV)
CTL: cytotoxic T cell
iDC: immature dendritic cell
IDS: interval debulking surgery
DC(s): dendritic cell(s)
DNA: deoxyribonucleic acid
EOT: end of treatment
FIGO: Fédération Internationale de Gynécologie et d’Obstétrique
GCIG: Gynecologic Cancer Intergroup
GCP: good clinical practice
GMP: good manufacturing practice
HLA: human leukocyte antigen
HGSOC: high-grade serous ovarian cancer
ICH: International Conference on Harmonization
OC-DC: autologous DCs pulsed with autologous oxidized tumor lysate (Vaccine)
OC: ovarian cancer
OS: overall survival
MS: mass spectrometry
NeoAg: neoantigen
PBMC: peripheral blood mononuclear cell
PDS: primary debulking surgery
PEPs: personalized peptides
PEP-DC: autologous DCs pulsed with personalized peptides (vaccine)
PET: positron emission tomography
RECIST: response evaluation criteria in solid tumors
SOC: standard of care
TAA: tumor-associated antigen
TCR: T cell receptor
TIL(s): tumor infiltrating lymphocyte(s)

## Good clinical practice

The procedures set out in this trial protocol, pertaining to the conduct, evaluation and documentation of this trial, are designed to ensure that all persons involved in the trial abide by Good Clinical Practice and the ethical principles described in the current revision of the Declaration of Helsinki. The trial will be carried out in keeping with local legal and regulatory requirements.
